# Clinical ethical practice and associated factors in healthcare facilities in Ethiopia: a cross-sectional study

**DOI:** 10.1186/s12910-022-00800-0

**Published:** 2022-06-18

**Authors:** Nebiyou Tafesse, Assegid Samuel, Abiyu Geta, Fantanesh Desalegn, Lidia Gebru, Tezera Tadele, Ewnetu Genet, Mulugeta Abate, Kemal Jemal

**Affiliations:** 1 Department of Public Health, College of Health Sciences, Kotebe University of Education, Addis Ababa, Ethiopia; 2 Human Resource Development Directorate, Ministry of Health Ethiopia, Addis Ababa, Ethiopia; 3 Ethiopian Public Health Association, Addis Ababa, Ethiopia; 4 Department of Nursing, College of Medicine and Health Sciences, Salale University, Fitche, Ethiopia

**Keywords:** Ethical, Clinical practice, Ethical committee, Healthcare workers, Ethiopia

## Abstract

**Background:**

Clinical ethical practice (CEP) is required for healthcare workers (HCWs) to improve health-care delivery. However, there are gaps between accepted ethical standards and CEP in Ethiopia. There have been limited studies conducted on CEP in the country. Therefore, this study aimed to determine the magnitude and associated factors of CEP among healthcare workers in healthcare facilities in Ethiopia.

**Method:**

From February to April 2021, a mixed-method study was conducted in 24 health facilities, combining quantitative and qualitative methods. Quantitative (survey questionnaire) and qualitative (semi-structured interviews) data were collected. For quantitative and qualitative data analysis, Stata version 14 and Atlas.ti version 7 were utilized. Multiple logistic regression and thematic analysis for quantative and qualitative respectively used.

**Results:**

From a total of 432 study participants, 407 HCWs were involved in the quantitative analysis, 36 participants were involved in five focus group discussions (FGDs), and eleven key informant interviews (KIIs) were involved in the qualitative analysis. The score of good CEP was 32.68%. Similarly, the scores of good knowledge and attitude were 33.50% and 25.31%, respectively. In the multiple logistic regression models, satisfaction with the current profession, availability of functional CECs, compassionate leaders, previously thought clinical ethics in pre-service education and good attitude were significant factors associated with CEP. Among these significant factors, knowledge, compassionate leaders, poor infrastructure, a conducive environment and positive attitudes were also determinants of CEP according to qualitative findings.

**Conclusions:**

The CEP in health care services in Ethiopia is low. Satisfaction with the current profession, functional CECs, positive attitude, compassionate leaders and previously thought clinical ethics were significant factors associated with CEP. The Ministry of Health (MoH) should integrate interventions by considering CECs, compassionate leadership, and positive attitudes and enhance the knowledge of health professionals. Additionally, digitalization, intersectoral collaboration and institutionalization are important for promoting CEP.

**Supplementary Information:**

The online version contains supplementary material available at 10.1186/s12910-022-00800-0.

## Background

Ethics is the application of moral rules and values to human activities. HCWs must possess not only the necessary skills and knowledge, but also be aware of the ethical and legal responsibilities that come with standard practices [1]. Clinical ethics has been founded on the framework of four moral principles of autonomy, beneficence, non-maleficence, and justice [2, 3].

Beneficence is the responsibility to prevent or eliminate harm while also promoting good by contributing to welfare and acting in the patient's best interests. Non-maleficence refers to a physician's obligation not to cause harm or adverse effects to a patient as a result of ineffective or absent care. Last but not least, justice entails distributing benefits, risks, and costs fairly, equitably, and appropriately, as well as treating patients with similar cases in the same way [4, 5].

The public has become increasingly concerned about HCWs’ ethical behavior. This is frequently manifested as complaints about unethical behavior and an increase in the use of legal action against healthcare providers [6].

A primary responsibility of practicing healthcare workers is to make decisions about patient care in a variety of settings. These choices entail more than just picking the best treatment or intervention [7]. The healthcare workers have an ethical obligation to [8] benefit the patient, (ii) avoid or minimize harm, and (iii) respect the patient's values and preferences. It has been demonstrated that a goal-oriented ethics educational program [2] improves learner awareness, attitudes, knowledge, moral reasoning, and confidence [3, 4].

Attending to ethical issues in clinical medications has changed in recent years as a result of heavy changes in both inward medicine and society. Among the many factors causing the increased prominence of ethics in medicine are unprecedented increases in scientific knowledge, expansion of the accessibility and efficaciousness of medical technologies, more patients and HCWs, new organizations in the provision of services, and increased pressure to contain tolls [9].

All types of healthcare workers have made significant contributions to Ethiopia's healthcare system. While the country has been praised for its improved health indicators, there has been a recent uptick in public outrage against health care professionals [10], as well as an increase in the number of complaints filed against health care workers [11].

Ethical principles aid in determining what is considered right or wrong at a given time in a given culture based on the action's perceived moral consequences [12]. Clinical ethics is concerned with healthcare workers' ethical responsibilities to their patients, colleagues, and society [13]. Ethics frequently prescribes a higher standard of conduct than the law [14]; what is legal is not always moral or ethical.

Many ethical difficulties relating to healthcare have arisen as a result of amazing developments in medical science, and they must be handled with high delicacy and professionalism in accordance with numerous medical ethics guidelines [15, 16]. As a result of extraordinary advances in medical technology, many ethical issues connected to healthcare have developed and have been addressed with great care and expertise in compliance with several medical ethics principles. This could be a result of both increasing public knowledge and healthcare providers' unethical behavior [16, 17].

Despite improvements in HCWs’ ethics understanding, there are still gaps between accepted ethical standards and inattentive knowledge provided in ethics courses, and health-care practitioners typically try to isolate attempts to use the knowledge in their routine clinical practice [16, 18]. The majority of articles deal with discussing the ethics of health care professionals with other health-care professionals. A reported course for health practitioners that used realistic scenarios to assist them in bridging the gap between theory and practice is an exception [18, 19]. The dynamic nature of the health professional-patient interaction, as well as the character of modern health practice commercialization, has had an impact on health profession practice. Negligence is thought to be the leading cause of patient misery. Unsatisfied patients are increasingly filing medical malpractice lawsuits [20, 21].

The recent rise in legal action against health care providers is a cause for serious worry. Economic, social, judicial, and professional considerations are to blame [22, 23]. Increasing media awareness of medical truths and fallacies, professional accountability, and patient rights in terms of information, decision-making, and result assessment are among these social elements. The media's coverage of the profession exacerbated the problem. Furthermore, in recent years, the debate between health professionals and patients has grown [22, 23]. Professional ethics has been incorporated into the educational curriculum of health professionals in many countries, and ethics committees and ethics specialists have been established. Ethics committees are the most important formal institutional mechanism for considering and resolving ethical dilemmas in health care institutions [24, 25].

Clinical ethics is the application of science and morality in medicine and the health sciences. Healthcare practitioners are expected to practice dignity, honesty, integrity, and responsibility, according to all laws, and refrain from engaging in or concealing any unethical, fraudulent, misleading, or deceptive action [26, 27].

There has been no attempt to teach ethics and law as separate courses integrated into the curriculum, even among other healthcare professionals [28]. It is necessary to determine the current basic knowledge and attitudes of healthcare practitioners to develop an ethical curriculum [29]. Few studies have been conducted to assess what is known and practiced in Ethiopia to better target educational efforts. According to one study, there was little evidence of CEP in Ethiopian health-care facilities [30]. As a result, the purpose of this study was to determine the level of CEP and associated factors among Ethiopian health care workers (HCWs).

### Research questions

1. What was the level of the clinical ethical practice in the healthcare facilities in Ethiopia?
2. What were the distinguishing features of clinical ethical practice in healthcare facilities in Ethiopia?
3. Which structural or institutional components affect clinical ethical practice in healthcare facilities in Ethiopia?

## Methods

### Study setting and period

This nationwide assessment was conducted at 24 health care facilities in Ethiopia. Recently, Ethiopia has 273,601 heath workers employed in public health facilities; of them, 181,872 (66.5%) were health professionals, and the remaining 91,723 (33.5%) were administrative staff [31].

Among health professionals, the highest three professional categories were nurses, health extension workers and midwifery, which accounted for 59,063 (21.59%), 41,826 (15%) and 18,336 (6.71%), respectively. The private health sector provides work opportunities for approximately 60,000 human resources for health in Ethiopia. There were 17,162, 3678 and 314 functional health posts, health centers and all types of hospitals, respectively, across all regions of the country that provide health services to the community; the hospitals were 22 tertiary hospitals, 73 general hospitals and 245 primary hospitals [31]. Although the Ethiopian health system shows improvement in quality health services, CEP services are limited. The study was conducted from February to April 2021. All methods were performed in accordance with relevant guidelines and regulations.

### Study design

An institution-based cross-sectional quantitative study triangulated with a qualitative approach (phenomenology design) was conducted [32]. Quantitative data were gathered through a structured questionnaire administered to the HCWs. The facility status towards CEP was assessed using a checklist.

Additionally, qualitative data were collected from KIIs (medical directors, chief executive officers (CEOs), quality focal persons, CRC focal persons, and quality directors) through an in-depth interview and FGDs (HCWs selected from each department).

### Study populations

The source population for this study was all HCWs who worked at primary, secondary and tertiary public hospitals in Ethiopia, whereas the study population was all HCWs who worked at the selected public hospitals in Ethiopia.

### Participant health facilities

For this study, 24 health care facilities were selected from 9 regions and 2 city administrations. Six tertiary, eleven secondary and seven primary hospitals were selected from all regions in Ethiopia. Two health care facilities were selected from each (Addis Ababa, Sidama, Afar, Benshangul Gumuz, Gambella), three health care facilities from Amhara, Oromia, Somali, SNNP and one health care facility selected from Harari and Dire Dawa. HCWs who had been working for more than six months in 24 health care facilities in Ethiopia were included. HCWs who had been on annual leave and transferred from other health care facilities that served less than six months were excluded.

For the qualitative study, the study participants were recruited from 24 health care facilities depending on their position and clinical experience using a purposive sampling technique. The KII participants were CEOs, medical directors, CRC focal persons, and quality focal persons. We invited them to participate in FGDs and in-depth interviews through invitation letters.

### Sample size determination and sampling procedure

The sample size was determined by using Epi-Info version 3.5.1 software [33] by a single population proportion formula with the assumptions of a 95% confidence level and 5% precision, taking a 50% proportion due to the lack of a previous study. A sample size of 435 was obtained after adding a 13% nonresponse rate. The sample size was proportionally allocated based on the number of HCWs per facility.

The sampling frame was prepared for each hospital by obtaining lists of HCWs from the directors and the human resource management office. Finally, 435 study subjects were selected from participating hospitals using a simple random sampling technique.

Purposive sampling was used to collect qualitative data, with 47 study participants interviewed. The trustworthiness of qualitative data was determined by considering plausible information gathered from participants through face-to-face interviews.

For the qualitative study, 36 participants were selected from health care facilities for FGDs (four FGDs contained 7 participants, and one FGD contained 8 participants). For KIIs, eleven KII participants (CEO, medical director, CRC focal person, and quality focal person) were selected from five healthcare facilities.

### Data collection tools, procedures and quality assurance

Data were collected using a standardized and pretested questionnaire. The questionnaire was adapted from different works in the literature and/or developed from other similar studies [34, 35]. The questionnaire contains sociodemographic characteristics, clinical ethical practice, knowledge and attitude of ethics, professional satisfaction, availability of clinical ethical committees (CECs), previous educational curriculum, and facilities auditing (an observational checklist) and was employed to observe and check the level of CEP (Additional file 1).

The tool's reliability test revealed a good estimate of the measurement: knowledge of CEP measured with 13 items and scale reliability coefficient (Cronbach's alpha) of 0.90, CEP measured with 12 items and scale reliability coefficient (Cronbach's alpha) of 0.83, and attitude towards CEP measured with 12 items and scale reliability coefficient (Cronbach's alpha) was 0.69. The overall scale reliability coefficient (Cronbach’s alpha) of all 37 items was 0.82. The questionnaire was prepared in the English language, translated into Amharic, Afan Oromo, and Somali languages and back-translated to English language-by-language experts. Data were collected using a self-administration questionnaire for HCWs.

Data were collected by 34 data collectors and supervised by 17 supervisors. Data collectors and supervisors were recruited based on their ability to speak Amharic language and fluent in each specific region language because they were recruited for data collection and supervision in regions with previous data collection experience.

The interviewers were health professionals with BSc and above and fluent speakers of Amharic language trained on the tools. The networks of the technical working group (TWG) were organized to enhance the data collection process. Three days of training was given for the data collectors and supervisors to adhere to the objective of the study. Additionally, COVID-19 prevention protocol training was given. The tools were also pretested (5% of the sample) in the health care facilities (non-selected facility). Based on the pretest findings, the tools were validated. During data collection, the tools were also checked by the investigators for completeness and consistency. In addition, the tools were coded during data collection and before entering the computer.

The qualitative data were collected through guiding questions in the Amharic language. A semi-structured interview guide was developed to assess CEP, awareness and factors associated with CEP. During KIIs and focus group discussions (FGDs), one modulator and reporter for each KII and FGD were recruited. The voices of the interviewed participants were recorded during the discussion and probing.

The outcome variable of CEP was measured using 12 items. Good practice (participants who scored > 75% and poor practice (participants who scored < 75% on practice-based questions) [36].

Participants who scored > 75% were labeled as having a favorable attitude and participants who scored < 75% were labeled as having an unfavorable attitude.

The level of knowledge and attitude were measured using 13 items each. Participants who scored > 75% were labeled knowledgeable, and those who scored < 75% were not knowledgeable [35].

Facility level CEP audit: measured with 15 items and reported as good score greater than 75% and poor if it is less than 75%.

### Data processing and analysis

Quantitative data were cleaned and entered into EPIDATA version 3 and STATA version 14 to obtain summary figures and percentages. Pearson’s chi-square and chi square tests of association were applied to look for differences. Multiple logistic regressions were performed to assess the strengths of the association. The analytic part was analyzed with their 95% confidence interval, and a two-tailed *p* value was calculated to identify the presence and strength of association. The categorical variables were dichotomized based on the cut-off point of the mean for binary logistic regression. Variables with a *p* value ≤ 0.2 in the binary analysis were included in a multivariable logistic regression analysis to control the confounding effect among the variables. Statistical significance was declared if the *p* value < 0.05. The satisfaction of health professionals with their current profession, the availability of a functional clinical ethics committee, compassionate leaders, and pre-service clinical ethics education were all covariates in the multiple logistic regression model.

The qualitative data were transcribed and translated from local languages to English. The qualitative analysis was facilitated by Atlas.ti version 7. The verbatim transcripts were separately verified against the interviewer's audio-recorded and analysis team members. The analysis team further guaranteed the consistency of each focus group and interview session. The teams also analyzed the interviewer's adherence to each transcript's probing questions and provided feedback and correction.

They read through each transcript again to reduce differences between individual codes and marked out the transcript that was non-specified to CEP through the agreement. Rigors were further assured by the study team, who was not being analyzed in the interviews or focus groups, independently auditing the coding process. Finally, the narrative was organized and integrated according to emerging themes and concepts set in the research objectives. The result was triangulated with quantitative findings at the interpretation level. A thematic analysis method was employed to report the qualitative study.

## Results

### Sociodemographic characteristics of health professionals

In the study, 407 HCWs participated, giving a response rate of 97%. During this study, 51.11% of the respondents were males, and approximately 45.45% of the respondents were aged below 30. Approximately 56.51% of the participants were married. Regarding profession, approximately 40% were nurses, and 20% were public health officers. In relation to work experience, approximately 53% of the participants had less than 5 years of experience. In this study, 58.97% of the respondents were happy with their current profession (Table 1).

**Table 1:** Sociodemographic characteristics of health professionals Ethiopia (n = 407), Ethiopia, 2021

Variables	Frequency (%)
Gender	
Male	208 (51.11)
Female	199 (48.89)
Age	
≤ 30	185 (45.45)
31–39	132 (32.43)
40–49	74 (18.18)
≥ 50	16 (3.93)
Marital status	
Married	230 (56.51)
Others*	177 (43.49)
Educational level	
Diploma	74 (18.18)
BSc	226 (55.55)
MSc/MPH	59 (14.50)
GP	31 (7.62)
Specialist	17 (4.18)
Job category/Profession	2 (0.49)
Nurse	161 (39.56)
Public Health	84 (20.64)
Physician	51 (12.53)
Midwives	48 (11.79
Pharmacy	27 (5.89)
Laboratory	21 (5.16)
Other^®^	15 (3.69)
Work experience in (years)	
1–5	214 (52.58)
5–10	114 (28.01)
≥ 10	79 (19.41)
Are you happy with the current profession/work?	
Yes	240 (58.97)
No	167 (41.03)

*Single, divorced and widowed :

^®^Anesthesia, Radiology, Physiotherapy, Psychiatry, and Environmental Health

### Facilities audit towards ethical practice

According to the facility audit, approximately 46%, 29% and 25% of the health care facilities were secondary hospitals, primary facilities and tertiary hospitals, respectively. From the total facilities, 70.83% were governed by the board of directors, and in 58.33% of the facilities, the board helped to decide clinical ethical issues while the case was raised; however, only 54.17% of health facilities had clinical ethical committees, 70.83% of facilities had no code of ethics for the departments, and 41.67% of the facilities also had no recording system or documentation. Regarding the empowerment of patients, 75% of facilities informed their patients of their rights and responsibilities, 66.67% of facilities encouraged the use of informed consent, and 66.67% of consent forms in the facilities were complete. Generally, the facilities status towards ethical practice was 54.17% (Additional file 2: Tables).

### Knowledge of health professionals towards CEP

Regarding knowledge of CEP, approximately 43% of the study participants knew the code of ethics for their respective professions; however, only 24.57% of them knew the fundamental principles of clinical ethics. In addition, only 13% and 16% of the respondents knew the types of ethical dilemmas and the meaning of preventive ethics, respectively. With respect to knowing medico-legal issues in health facilities and proclamation for clinical practice in Ethiopia, only 14% and 15.3% were knowledgeable, respectively. Overall, only 34% of the respondents were above the 75% about the knowledge of component of CEP (Table 2).

**Table 2:** Knowledge of health professionals towards clinical ethical practice (n = 407), Ethiopia, 2021

S.no	Statements of clinical ethical practice knowledge of health professionals	Yes, n (%)	No, n (%)
1.	Do you know your professional codes of conduct?	175 (43)	232 (57)
2.	Do you know code of ethics?	100 (24.57)	307 (75.43)
3.	Do you know the fundamental principles of ethics?	51 (12.53)	356 (87.47)
4.	Do you know the types of ethical dilemma?	65 (15.97)	342 (84.03)
5.	Do you know what the meaning of preventive ethics?	92 (22.60)	315 (77.40)
6.	Do you know the meaning of patients’ rights?	76 (18.72)	330 (81.28)
7.	Do you know the benefit of preventive ethics?	83 (20.39)	324 (79.61)
8.	Do you know the application or purpose of code of ethics?	71 (17.44)	336 (82.56)
9.	Do you know the roles and duties of clinical ethical committee in your facility?	80 (19.66)	327 (80.34)
10.	Do you know the purpose of consent taking from the patient?	61 (14.99)	346 (85.01)
11.	Do you know medico-legal issue in the health care facility?	57 (14.00)	350 (86.00)
12.	Do you know proclamation stated for clinical ethical practice in Ethiopia?	62 (15.23)	345 (84.77)
13.	Do you know legal and social accountability with regard to clinical ethical practice in Ethiopia?	57 (14.00)	350 (86.00)
	**Knowledgeable (> 75%)**	**136 (33.50)**	**270 (66.50)**

Bold showed the average results and this is to emphasis the findings

### Clinical ethical practice of health professionals

With respect to the regular application of ethical principles while providing health care services, only 39% practiced ethical principles regularly, and 54% took consent before the provision of service.

Regarding confidentiality and privacy, 66% and 65% of the respondents’ protected patient confidentiality and privacy, respectively; however, only 44.47% of the respondents affirmed disclosing an ethical breach when it appeared. In addition, 52.58% practice codes of ethics of their respective professions. Generally, 32.6% of the respondents scored ≥ 75% towards CEP (Table 3).

**Table 3:** Clinical ethical practice of health professionals (n = 407) Ethiopia, 2021

S.no	Statements of clinical ethical practice of health professionals	Yes, n (%)	No, n (%)
1.	Do health professionals regularly practice principles of ethics in service provision in your facility?	155 (38.08)	252 (61.92)
2.	Do health professionals take consent from patients within your profession before service provision?	219 (53.81)	188 (46.19)
3.	Do health professionals respects patients’ rights during service provision	264 (64.6)	143 (35.14)
4.	Do health professionals keep confidentiality of the patients during service provision?	268 (65.85)	139 (34.15)
5.	Do health professionals maintain privacy of the patients during service provision?	264 (64.86)	143 (35.14)
6.	Do health professionals apply preventive ethical procedures to resolve ethical dilemma?	230 (56.51)	177 (43.49)
7.	Do you disclose ethical breach during service provision?	181 (44.47)	226 (55.53)
8.	Do health professionals involve family in treatment decision of the patients?	200 (49.14)	207 (50.86)
9.	Do health professionals involve patients in treatment decisions?	224 (55.04)	183 (44.46)
10.	Do health professionals use clinical ethics consultation for prevention of medical errors?	230 (56.51)	177 (43.49)
11.	Have you practiced your professional code of ethics in service provision?	225 (55.28)	182 (44.72)
12.	Do health professional use code of ethics for prevention of medical errors?	214 (52.58)	193 (47.42)
	**Clinical Ethical Practice (participants who scored ≥ 75% on practice based questions)**	**133 (32.68)**	**274 (67.32)**

Bold showed the average results and this is to emphasis the findings

### Attitudes of health professionals towards CEP

The majority of the respondents, with an 86.73% rate, believed that ethical conduct is important only to avoid legal action, and only 28% assumed that patients are always told if something goes wrong. In contrast, 82.56% of the respondents thought HCWs should do what is best irrespective of the patients’ opinion, and 27.52% believed close relatives must always be informed about a patient’s condition. Regarding health care laws and proclamations, 13% of the respondents thought that the presence of law allows abortion to be performed; therefore, HCWs cannot refuse to do an abortion for clients. Moreover, 68% of the respondents thought children should never be treated without the consent of their parents or guardians. In general, 25.31% of the respondents scored above the mean score for CEP (Table 4).

**Table 4:** Attitudes of health professionals towards ethical clinical practice (n = 407), Ethiopia, 2021

S.no	Statements of ethical clinical practice attitude of health professionals	Yes, n (%)	No, n (%)
1.	‘Ethical conduct is only important to avoid legal action’	253 (86.73)	54 (13.27)
2.	The patient’s wishes must always be adhered to’	70 (17.20)	337 (82.80)
3.	‘The patient should always be told if something goes wrong’	114 (28.01)	293 (71.99)
4.	Confidentiality cannot be kept in modern care and should be abandoned’	321 (78.87)	86 (21.13)
5.	The health professionals should do what is best irrespective of the patients opinion’	336 (82.56)	71 (17.44)
6.	Patients only need to consent for operations not for tests or medications’	311 (76.41)	96 (23.59)
7.	Close relatives must always be informed about a patient’s condition’	112 (27.52)	295 (72.48)
8.	Children [except in an emergency] should never be treated without the consent of their parents or guardians’	275 (67.57)	132 (32.43)
9.	health professionals should refuse to treat patients who behave violently’	281 (69.04)	126 (30.96)
10.	The law allows abortion to be performed, therefore health professionals cannot refuse to do an abortion for a patient’	50 (12.29)	357 (87.71)
11.	A patient who wishes to die should be assisted in doing so no matter what their illness’	121 (29.73)	286 (70.27)
12.	‘A patient who refuses to be treated on religious or other grounds should be told that they need to find another health professional with their beliefs or accept the treatment offered	109 (26.78)	298 (73.22)
	**Attitude (scored > 75%)**	**103 (25.31)**	**304 (74.69)**

Bold showed the average results and this is to emphasis the findings

### Variables related to CEP

In relation to a source of information on ethical clinical practice, 43.24% and 43% of the respondents gained information from media and reading, respectively. In addition, only 21.38% of the respondents considered CEP in the educational curriculum to be adequate. It was also found that only 23.46% of the respondents took ethical clinical practice training. During their clinical practice, the dominant ethical dilemma that health professionals encountered was quitting medical practice, with a 47.54% rate of all dilemmas followed by discharge against medical advice and religious and cultural issues. The main causes identified for ethical malpractice of HCWs were workload, with a 64.29% rate of all causes, followed by lack of knowledge and unaccountability. However, 58.97% of the respondents were satisfied with their current profession (Additional file 2: Tables).

### Factors associated with CEP

In the multiple logistic regression model, health professionals who were satisfied with their current profession were 1.56 times [AOR (adjusted odds ratio) = 1.56; 95% CI = (1.01–2.43)] more likely to practice CEPs than those who were not satisfied with their profession. Similarly, in public hospitals, when there was availability of functional ECCs, HCWs practiced CEP 1.71 times [AOR = 1.71; 95% CI = (1.10–2.66)] more likely than when there was no CEC. In public hospitals, compassionate leaders were 1.92 times [AOR = 1.92; 95% CI = (1.23–23.00)] more likely to practice CEPs than other leaders. HCWs who previously thought clinical ethics in pre-service education were 1.83 times [AOR = 1.83; 95% CI = (1.13–2.98)] more likely to practice CEPs than others. However, sex, age, educational level, knowledge of ethical clinical services, previous training on ethical clinical services and workload had no significant association with CEP (Table 5).

**Table 5:** Multiple logistic regressions towards ethical clinical service in Ethiopia (n = 407), 2021

Variable	Ethical clinical practice		COR (95% CI)	*p* value	AOR (95% CI)	*p* value
	Good	Poor				
Sex						
Male	76	132	1.43 (0.94–2.18)	0.90	1.39 (0.91–2.15)	0.118
Female	57	142	1.0		1.0	
Age						
≤ 30	66	119	0.87 (0.54–1.39)	0.567	0.87 (0.53–1.43)	0.565
31–39	43	89	0.67 (0.36–1.21)	0.183	0.67 (0.36–1.27)	0.151
40–49	20	54	0.60 (0.19–1.94)	0.394	0.76 (0.21–2.74)	0.402
≥ 50	4	12	1.0			
Educational level						
High	40	67	1.33 (0.84–2.11)	0.228	1.26 (0.78–2.03)	0.116
Low	93	207	1.0		1.0	
Years of experience						
> 10 years	55	138	0.69 (0.45–1.06)	0.088	0.66 (0.43–1.02)	0.090
< 10 years	78	136	1.0		1.0	
Satisfaction with their current profession						
Yes	88	45	1.57 (1.02–2.42)	**0.040**	**1.55 (1.01–2.39)**	**0.045**
No	152	122	1.0		1.0	
Knowledge						
Knowledgeable	49	87	1.24 (0.81–1.93)	0.319	1.20 (0.76–1.91)	0.525
Not Knowledgeable	84	186	1.0		1.0	
Availability of functional ECCs						
Yes	59	82	1.87 (1.22–2.87)	**0.004**	**1.76 (1.14–2.73)**	**0.010**
No	74	192	1.0		1.0	
Source of information						
Mass media						
Yes	63	113	1.28 (0.84–1.94)		1.26 (0,82–1.91)	
No	70	161	1.0		1.0	
Leadership style						
Compassionate	68	93	2.05 (1.35–3.14)	**0.001**	**1.93 (1.26–2.98)**	**0.003**
Non-compassionate	64	180	1.0		1.0	
College and University						
Yes	49	86	1.28 (0.83–1.97)	0.273	1.04 (0.65–1.64)	0.268
No	84	188	1.0		1.0	
Thought clinical ethics in pre-service education						
Yes	95	38	1.83 (1.13–2.98)	**0.014**	**1.72 (1.05–2.82)**	**0.031**
No	225	49	1.0		1.0	
Encountered ethical clinical problem						
Yes	31	57	1.16 (0.70–1.90)	0.565	1.11 (0.67–1.87)	0.649
No	102	217	1.0		1.0	
Took clinical ethics training						
Yes	37	58	1.42 (0.88–2.29)	0.148	1.37 (0.84–2.23)	0.201
No	96	214	1.0		1.0	
Is the clinical ethical training adequate?						
Yes	27	48	1.08 (0.63–1.85)	0.790	0.92 (0.52–1.62)	0.972
No	81	155	1.0		1.0	
Workload						
No	46	99	0.95 (0.61–1.46)	0.801	0.91 (0.78–1.23)	0.959
Yes	86	175	1.0		1.0	
Attitude towards CEP						
Favorable	21	112	1.95 (1.04–3.65)	**0.036**	**1.92 (1.02–3.66)**	**0.044**
Unfavorable	24	250	1.0		1.0	

Bold indicates the signficance variables

## Qualitative findings

### Demographic characteristics of qualitative participants

In the qualitative study, a total of 47 individuals were involved in FGDs and KIIs. Of these, 11 were in the KIIs and 36 were in the FGDs. Of the KIIs group, 03 were within the age group of 25–30, whereas among the FGDs. Most FGD discussants were females (26); however, in the KIIs, nearly half (05) of the participants were males. Marital status was only asked in the FGD participants so that the majorities, 25 individuals, were married. Concerning their educational status, 17 individuals who were involved in the FGDs had a diploma level of education, whereas 29 had BSc and above.

In the KIIs, 03 CEOs, 04 quality directors, 02 CRC focal persons and 02 medical directors were involved. Professionally, 03 were nurses, 02 were medical doctors, and the remaining few were health officers, pharmacies and midwiferies. Regarding their service years with the current position (during the date of data collection), 03 had only one year and less work experience, and 03 had between two and three years of work experience (Table 6).

**Table 6:** Demographic characteristics of the FGD and IDI participants (n = 47), Ethiopia, 2021

FGD participants		IDIs participants	
Characteristics	Number of participants	Characteristics	Number of participants
Age		Age in years	
< 25	06	< 25	04
25–30	15	25–30	03
31–35	10	31–35	02
> 35	05	> 35	02
Gender		Gender	
Female	26	Female	05
Male	10	Male	06
Marital status		Position	
Married	25	Chief executive officer	03
Single	18	Quality service director	04
Widowed	2	CRC focal person	02
Divorced	1	Medical director	02
Educational status		Profession	
Diploma	17	Nurses	03
BSc and above	29	Medical doctor	02
Position		Health officer	02
Yes	10	Pharmacy	01
No	26	Midwifes	01
		Laboratory	02

The health care system evolved through different stages of development, and remarkable achievement was achieved. From KIIs and FGDs, we found six main themes, namely, low awareness, low practice, positive attitude, professional satisfaction, challenges and facilitators (Additional file 3: Qualitative results).

#### Low awareness of CEP

The awareness of CEP in 24 public health facilities showed significant gaps. There are limitations on the awareness of CEPs, proclamation on the code of ethics, and respectful care practices.

The majority of the study participants described that there was a problem with knowledge about CEP. The study participants from KII elaborated as follows:

*“---there is lack of knowledge towards preventive ethics and ethical dilemmas.” (KII 2)*

Awareness of the proclamations of ethical codes of conduct is the core for better implementation of CEP. However, the KII participant depicted that there are gaps among health professionals working in public health facilities.

*“Most of the health professionals working in this hospital are not aware of the types and the detailed parts of the proclamation stated for clinical practice, and even some of the professionals are not familiar with the core principles of CEP.” (KII 4)*

In addition, the FGD participant also added the following:

*“We are not so familiar with the proclamation released regarding HCWs in Ethiopia and Ministry of Health (MoH) needs to intervene in such area for better implementation of CEP.” (FGD, Participant (P8)*

#### Lower practice of clinical ethics

There is a lack of clinical ethics in service delivery in public health facilities. Accordingly, the KII participants indicated gaps in practice and the implementation of clinical ethics practice.

*“There are gaps in the practice of clinical ethical practice among health care workers in public health facilities in Ethiopia.” (IDI 3)*

*“The implementation of preventive ethics in our facility is not as expected if you take, for example, when ethical dilemma encountered at the workplace there is no way of discussion by the well-organized team.” (IDI 6)*

#### Attitudes towards CEP

The attitude towards CEP of the health professional is the main determinant for better implementation of service delivery. The KIIs participants described as follows:

*“Positive attitude toward CEP is principal in all hospitals, and the health workers are cooperative and understand the patient’s complex issues.” (KII 4)*

*“At this time, there is good awareness due to compassionate and respectful care training; at least, everybody knows the fundamental principles of ethics and creates a positive attitude towards the CEP.” (KII 5)*

#### Professional satisfaction

The professional satisfaction of health professionals is essential for the better performance of CEPs. The main factors affecting professional satisfaction were enhanced by the implementation of *CEP*. The KII and FGD participants added that:

“….I think, professional interest and satisfaction is instrumental for better performance and effectiveness of the service provision. However, in our country the basis for selection of profession entirely dependent on score of university entrance exam… I think this is the major problem for our health care ineffectiveness that led to professional dissatisfaction.” (IDI4)

“….At least at this time, there is situation that makes you satisfied with your current profession, one of this is from patient perspectives, see when your patient heals and recovered from the illness that will be the source of satisfaction” (FGD3, P4)

## Discussion

This study aimed to assess CEP and associated factors among HCWs working in public health facilities in Ethiopia. Our study depicted that 133 (32.7%) had good CEP, which indicates that most of the HCWs had poor CEP practices. Similarly, the qualitative theme findings showed gaps in CEP among HCWs. Our finding is consistent with studies conducted in Addis Ababa [30], Nigeria [37] and Pakistan [35]. Furthermore, the facility structural component (facility audit) of CEP in this study was 54%. This also signifies that CEP received less attention in the public health facilities in Ethiopia.

The findings of this study identified that health professionals’ satisfaction with their current profession, availability of functional ECCs, previously thought clinical ethics in pre-service education, good attitude and compassionate leadership style were significantly associated with CEP. However, sex, age, educational level, knowledge of ethical clinical services, previous training on ethical clinical services and workload had no significant association with CEP.

Those health professionals who were satisfied with their current profession were 1.56 times more likely to practice CEPs than those who were not satisfied with their profession. Similarly, the qualitative finding depicted that professional interest and satisfaction are core for better performance and effectiveness of the service provision. A consistent finding was also reported from Bolikhamsai Province, Lao PDR, revealing that health professionals were satisfied with their job, excluding their salary [38].

Furthermore, a similar finding reported from Iran showed that professional satisfaction of HCWs improves the ethical climate at work [39]. However, contrary qualitative finding reported in this study indicated that in our country the basis for selection of profession entirely dependent on score of university entrance exam… this the major problem for our health care ineffectiveness that led to dissatisfaction towards the professions. The possible reason for this difference could be due to recent initiations of motivated, competent and compassionated health workforce policy implementation by the Ministry of Health in Ethiopia.

According to the findings, HCWs at public health facilities with functional ECCs were 1.71 times more likely to have good CEP than those in facilities without functional ECCs. This could be related to the recent development of a Clinical Ethics Consultation Service (CECS) with the goal of improving health care providers' ability to deal with ethical dilemmas and reducing decisional clinical disputes.

Similarly, the facility audit in this study indicated that approximately 54% of the facilities have CEC. A comparable finding reported by *Althobaiti and his colleagues in 2021* from the Kingdom of Saudi Arabia (KSA) [40] depicted that CEC is a higher command level for analyzing and resolving ethical conflicts, as well as considering and resolving legal medical dilemmas. However, another systematic review revealed that CEC supports HCWs in managing controversial clinical ethical issues, but their impact on clinical practice is still unclear [41]. This variability could be due to differences in setting, method and HCW parameters.

Those health professionals who previously thought clinical ethics in pre-service education were 1.72 times more likely to practice good CEP than the others. A similar finding reported from the USA showed that grassroots medical ethics education emphasizes experiential learning [42]. In addition, a study performed in Turkey using Creswell’s six-step qualitative data analysis showed that third-grade medical students emphasized that discussions about movies, books, and case studies are beneficial [43]. In addition, only 21% of HCWs thought the clinical ethics integration in medical education was adequate, while others thought the given (one credit hour) was insufficient to teach and incorporate it with CEP.

The odds of having good CEP among HCWs with a favorable attitude were 1.92 times the odds of having an unfavorable attitude. Furthermore, the qualitative study identified attitude as one of the factors associated with CEP. This could be because the attitudes of health-care personnel influence what they do, and those who have a positive attitude are more likely to act positively [44].

HCWs who reported a compassionate leadership style in public health facilities were more likely to perform good CEPs than those not reported. This could be because it benefits health care workers’ well-being and patient outcomes by fostering integrative thinking and prosocial motivation [45]. In addition, the qualitative findings indicated that leaders have a supportive and practical role in CEP.

In the multiple logistic regressions, sex, age, educational level, knowledge of CEP, and previous training on ethical clinical service were not associated with CEP. This could be because of the similarity in the socioeconomic status of the HCWs. In contrast to our findings, several studies reported that gender [46], age [30], educational level [47], knowledge level [48], and previous training on CEP [14] were significantly associated with CEP. In addition, workload was not significantly associated with CEP. A consistent finding was reported in another study performed among Iranian nurses [49]. However, another study reported that workload among HCWs in Addis Ababa, Ethiopia, was significantly associated with CEP [50].

### Strengths and limitations of the study

This study covered all health professionals from public healthcare facilities throughout the country and was likely to ensure representativeness. In addition, the study employed a mixed study design using both quantitative and qualitative approaches. Due to the cross-sectional nature of this study, establishing a true cause and effect relationship between CEP and associated factors would be impossible.

## Conclusions

The CEP in health care services in Ethiopia is low. Satisfaction with the current profession, CEC, compassionate leaders and previously thought clinical ethics were significant factors associated with CEP. According to the qualitative findings, low awareness, low CEP, positive attitude, professional satisfaction, challenges and facilitators are also determinants of clinical ethical practice.

It is relevant to include CEP capacity building initiative to the continuous professional development (CPD) of HCWs. The MoH should integrate interventions by considering CECs, compassionate leadership, and positive attitudes and enhance the knowledge of health professionals. Additionally, digitalization, intersectoral collaboration and institutionalization are important for promoting CEP. Further studies warranted towards the structural determinants of CEP in the public health facilities in Ethiopia.

## Supplementary Information

**Additional file 1.** Questionnaire, structured questionnaire for HCWs.

**Additional file 2.** Tables.

**Additional file 3.** Qualitative results.

## Data Availability

The datasets generated and/or analyzed during the current study are not publicly available. Sharing of data was not included in the approval from the ethics committee but is available from the corresponding author on reasonable request.

## Abbreviations

CEP: Clinical ethical practice
CEC: Clinical ethical committee
EMoH: Ethiopian Ministry of Health
HCWs: Health care workers
HCFs: Health care facilities
HECs: Health Ethics Committee
KIIs: Key informant interviews
CEOs: Chief executive officers
TWG: Technical working group
FGDs: Focus group discussions
AOR: Adjusted odds ratio
