# Supplementary material for: Clinical ethical practice and associated factors in healthcare facilities in Ethiopia: a cross-sectional study
Source: BMC Med Ethics. 2022 Jun 18;23:61. doi: 10.1186/s12910-022-00800-0 (PMC9206399; doi:10.1186/s12910-022-00800-0)
Supplement: Supplementary file 1 — Additional file 1. Questionnaire, structured questionnaire for HCWs. [file 12910_2022_800_MOESM1_ESM.docx]

**Clinical Ethical Practice and associated factors in the healthcare facilties in Ethiopia, 2021**

**Instructions: Write your response on the space provided for closed ended questions and Encircle your Response for close ended questions**

| **Part I: Sociodemographic Characteristics of Health professionals** | | | |
| --- | --- | --- | --- |
| **Q. number** | **Questions** | **Response** | |
|  | What is your sex? | 1. male  2. Female | |
|  | How old are you? | 1.________ years | |
|  | What is your religion? | 1. Orthodox  2. Muslim  3. Protestant  4. others (Specify) __________ | |
|  | To which ethnic group do you belong? | 1. Amhara  2. Oromo  3. Tigrie  4. Guragie  5.Others(specify) __________ | |
|  | What is your marital status? | 1. Single  2. Married  3. Divorced  4. Widowed  5. Others ( Specify)________________ | |
|  | What is your current educational status? | 1. Diploma (LevelIV)  2. BSc  3. MSc /MPH  4. GP  5. Specialty  6. Subspecialty  7. PhD  8. Others ( Specify)________________ | |
|  | What is your current Profession? | 1. Medical Doctor  2. Health Officer  3. Nurse  4. Midwife  5. Anesthesia  6. Radiography  7. Laboratory  8. Pharmacy  9. Physiotherapy  10. Others (specify) ___________ | |
|  | At which department are you working? | Hospital | Health Center |
|  |  | 1. OPD 2. Surgical 3. Gyn/Obs 4. Radiology 5. Laboratory 6. Pharmacy 7. Emergency 8. Other | 1. OPD 2. Delivery 3. Laboratory 4. Pharmacy 5. Emergency 6. Other |
|  |  |  | |
|  | How long have you been in this profession? | 1. __________years | |
|  | Monthly salary | 1.Cash(in Birr)= _______ | |

| **Part V:- Factors Associated with Clinical Ethical Practice** | | | |
| --- | --- | --- | --- |
|  | What is your source of information about health professional ethics? (Multiple response ) | 1. Mass media 2. From college /university 3. From hospital management 4. From reading 5. Others ( specify )______________________ |  |
|  | How do you see the professionals’ ethics course in your previous educational curriculum? | 1. Adequate 2. Not adequate 3. could not remember whether or not it was taught |  |
|  | Is there clinical ethics committee in your institution? | 1. Yes 2. No |  |
|  | If answer to # 3 is yes do you know the role of clinical ethics committee? | 1. Yes 2. No |  |
|  | How did you encounter health professionals’ clinical ethical problem ? | 1. on daily basis 2. rarely 3. not at all |  |
|  | In your career have you been taken clinical ethics training? | 1. Yes 2. No |  |
|  | If answer to question # 6 is yes do you think the training is sufficient? | 1. Yes 2. No |  |
|  | How you frequently encountered ethical dilemmas? | 1. on daily basis 2. rarely 3. not at all |  |
|  | Which ethical dilemma encountered in service provision? Multiple answers possible | 1. discharge against medical advice 2. religious/cultural issues 3. truth telling 4. conflict of interest 5. end of life issues 6. others (specify ) |  |
|  | Why do you think that health care workers perform ethical malpractice unethically?(multiple response ) | 1. Due to work overload 2. Due to unaccountability 3. Due to negligence 4. Due to lack of knowledge 5. Due to poor legal action 6. Others (specify )________________ |  |
|  | Are you happy within your current profession? | 1. Yes 2. NO |  |
| **11** | If # 10 is the answer is no why? | 1. Overload  2. Not thought with interest  3. Lack of training  4. Low salary and other incentives  5. absence of Professional care  6. presence of professional risk  7. others ( Specify) _____________ |  |
| **12** | Is the leadership style in your facility compassionated? | 1. Yes 2. No |  |

^1^
