# Supplementary material for: Clinical ethical practice and associated factors in healthcare facilities in Ethiopia: a cross-sectional study
Source: BMC Med Ethics. 2022 Jun 18;23:61. doi: 10.1186/s12910-022-00800-0 (PMC9206399; doi:10.1186/s12910-022-00800-0)
Supplement: Supplementary file 2 — Additional file 2. Tables. [file 12910_2022_800_MOESM2_ESM.docx]

| **S.no** | **Facilities status towards ethical Practice** | **Yes, n (%)** | **No, n(%)** |
| --- | --- | --- | --- |
|  | **Does the facilities** |  |  |
|  | Have Clinical ethical \|committee? | 13(54.17) | 11(45.83) |
|  | have Manual on code of ethics per each department? | 7(29.17) | 17(70.83) |
|  | have board for leading and support of the facility? | 17(70.83) | 7(9.17) |
|  | board help to decide clinical ethical issues during the cases arises | 14(58.33) | 10(41.67) |
|  | provide awareness creation on application of prevent | 10(41.67) | 14(58.33) |
|  | have a reporting system on ethical breaches | 13(54.17) | 11(45.83) |
|  | Does the facility have legal personnel when there were ethical breaches | 12(50.00) | 12(50.00) |
|  | have a recording system or documentation | 14(58.33) | 10(41.67) |
|  | encourage the use of informed consent form? | 16(66.67) | 8(33.33) |
|  | have expert on ethical dilemma resolution? | 17(70.83) | 7(9.17) |
|  | inform patients’ rights and responsibilities? | 18(75.00) | 6( 25.00) |
|  | have a mechanisms \|for receiving appeals | 16(66.67) | 8(33.33) |
|  | consent form complete? | 16(66.67) | 8(33.33) |
|  | **Facility status towards ethical clinical practice (above the mean score )** | **13( 54.17)** | **11(45.83)** |

Table 1A: Facilities status towards Clinical Ethical Practice in Healthcare facilities in Ethiopia, 2021(n= 24)

**Table 2A: Description of factors related with ethical clinical practice, (n= 407), Ethiopia, 2021**

| **Variables** | **Frequency** | **Percentage** |
| --- | --- | --- |
| **Source of ethical clinical practice information** |  |  |
| Mass media | 176 | 43.24 |
| College and university | 135 | 33.17 |
| Hospital/health center leadership | 161 | 39.75 |
| Reading | 175 | 43.00 |
| Adequacy of ethical clinical practice in educational curriculum | 87 | 21.38 |
| Encountered ethical problem during clinical practice | 88 | 21.62 |
| Took ethical clinical practice training | 95 | 23.46 |
| Adequacy of ethical clinical practice training | 75 | 24.12 |
| **frequently encountered ethical dilemmas** |  |  |
| Quit medical service | 193 | 47.54 |
| discharge against medical advice | 117 | 28.75 |
| religious/cultural issues | 116 | 28.57 |
| truth telling | 114 | 28.01 |
| conflict of interest | 91 | 22.36 |
| end of life issues | 79 | 19.51 |
| **Reasons for ethical malpractice of health professional’s** |  |  |
| work overload | 261 | 64.29 |
| Unaccountability | 84 | 20.64 |
| Negligence | 105 | 25.80 |
| lack of knowledge | 112 | 27.52 |
| poor legal action | 93 | 22.85 |
| **Satisfied within your current profession** | 240 | 58.97 |
