# Supplementary material for: Clinical ethical practice and associated factors in healthcare facilities in Ethiopia: a cross-sectional study
Source: BMC Med Ethics. 2022 Jun 18;23:61. doi: 10.1186/s12910-022-00800-0 (PMC9206399; doi:10.1186/s12910-022-00800-0)
Supplement: Supplementary file 3 — Additional file 3. Qualitative results. [file 12910_2022_800_MOESM3_ESM.docx]

## Challenges of clinical ethical Practice

The main challenges for better implementation of CEP are infrastructure, lack of documentation, behaviors of the patients and lack of commitment. The FGD participant stated as follows:

*“I have an opportunity to observe the situation in both hospital and primary health care. I think all health professionals are graduated and well oriented from institutions. They promised to help their community honestly. However, sometimes the service receivers make them upset. There might be shortage of inputs for services.” (FGD6, P6)*

*“There might be conflict between the health profession and the patient. There were ethical malpractices. The big gap on CEP is lack of documentation about their performance, unsatisfactory and non-uniformity salary, and lack of interest on the profession.” (FGD4, P3)*

## Facilitators of CEP

Most of the participants recommended that digitalization is a key component to handle ethical malpractice and enhance the provision of health service delivery. The KII participant explained as follows:

*“Digitalization of service delivery is essential to our health care system. If this is implemented to our hospital, there will be workload reduction and efficient workflow and ultimately ending with minimal ethical malpractice.” (KII 6)*

Ethical committees are the structural component of clinical practice. There are differences between healthcare facilities with regard to the availability and functionalities of CECs. The FGD and KII participants stated as:

*“The role of CEC is indispensible. In our hospital, this committee is used to resolve ethical dilemmas and to manage medical errors. ” (FGD5, p4)*

*“Professional discussions were the source of solutions in hospitals and enabled health care workers to resolve several clinical ethical issues.” (KII 3)*

The support of leadership is central to the successful implementation of CEPs in healthcare facilties. The FGD and KII participants explained as follows:

*“Compassionate leaders are crucial for better implementation of CEPs. If you take, for example, in our hospital, the leaders have a supportive and practical role in CEP” (FGD 2, P3)*

*“I think* g*ood leadership practice is the principal for clinical ethical practice, and it needs operationalization to all aspects of the hospital working environment.” (KII 2)*
